# Anti-Cyclic Citrullinated Peptide Antibody and Periodontal Status in Rheumatoid Arthritis Patients

**DOI:** 10.12669/pjms.344.15007

**Published:** 2018

**Authors:** Wan Majdiah Wan Mohamad, Soh Ke Jia, Wan Syamimee Wan Ghazali, Haslina Taib

**Affiliations:** 1 Wan Majdiah Wan Mohamad, School of Dental Sciences, University Sains Malaysia, 16150 Kubang Kerian, Kelantan, Malaysia; 2 Soh Ke Jia, School of Dental Sciences, University Sains Malaysia, 16150 Kubang Kerian, Kelantan, Malaysia; 3 Wan Syamimee Wan Ghazali, School of Medical Sciences, University Sains Malaysia, 16150 Kubang Kerian, Kelantan, Malaysia; 4 Haslina Taib, School of Dental Sciences, University Sains Malaysia, 16150 Kubang Kerian, Kelantan, Malaysia

**Keywords:** Rheumatoid arthritis, Gingivitis, Periodontitis, Anti-cyclic citrullinated antibodies

## Abstract

**Objectives:**

Studies have shown that periodontal disease and Rheumatoid Arthritis (RA) shared similar pathogenesis. Anti-Cyclic Citrullinated Peptide anibodies (anti-CCP) has recently been used for diagnosis of RA. Thus, this study aimed to assess the levels of anti-CCP antibodies and periodontal status in RA patients.

**Methods:**

Forty four RA patients were included in this study. The blood samples were analysed for anti-CCP levels. Plaque Score (PS), Gingivitis Score (GS), Probing Pocket Depth (PPD) and Clinical Attachment Loss (CAL) were recorded for assessment of periodontal status. Relevant clinical information was obtained from medical records.

**Results:**

Mean anti-CCP level was 180.8 ± 290.3 Unit/ml. The results showed that 27.3% patients had poor oral hygiene (PS >60%; mean anti-CCP 84.22 ± 167.51 Unit/ml), 52.3% had generalized gingivitis (mean anti-CCP 145.07 ± 269.17 Unit/ml), and 20.5% had mean CAL of >3mm (mean anti-CCP 56.81 ± 119.02 Unit/ml). None of patients presented with deep PPD > 4mm. The levels of anti-CCP showed no significant association with periodontal status (*p*=0.27).

**Conclusion:**

Most RA patients were positive for anti-CCP antibodies and presented with generalized gingivitis. Oral hygiene education should be reinforced in RA patients to prevent further progression of periodontal disease. Nevertheless, studies with larger sample size should be carried out to obtain more conclusive findings.

## INTRODUCTION

Rheumatoid Arthritis (RA) is an inflammatory disease characterised by chronic inflammation of the joints that leads to cartilage degradation, bone erosion and physical disability.^1^ It primarily affects the joint and may cause devastating complications.^2^ RA affects 1.0% of the world population with a female to male ratio of 3:1 and has a peak incidence of onset in women in the third and fourth decades of life.^3^ The prevalence of RA in Malaysia is 0.5%.^4^ The exact aetiology of RA remains unknown. However, there is possible postulation that genetic and environmental factors favour the development of the disease. Expression of Major Histocompatibility Complex (MHC) class II human leucocyte antigen-DRB1 (HLA-DRB1) allele has been found in RA patients and combination of HLA-DRB1 Shared Epitope (SE) alleles and smoking was known to be the risk factor for developing RA.^5^,^6^ Up to date, periodontitis has been found to be a risk factor for RA. Epidemiological studies have shown that patients with periodontitis have higher probability of developing RA.^7^

Periodontitis is an inflammatory disease of the tooth-supporting tissues caused by specific microorganisms, mostly aerobic and gram-negative bacteria and leads to progressive destruction of the periodontal ligament and alveolar bone.^8^ The primary clinical features of periodontitis include gingival inflammation, periodontal pocketing, and alveolar bone loss which may end up with loosening of teeth and tooth loss.

### Porphyromonas Gingivalis

the main leading pathogen in periodontitis^9^ posseses a unique microbial enzyme known as Peptidylarginine Deiminase (PAD), which causes a specific post-translational modification, in which arginine residues are converted into citrulline residues through a process called protein citrullination.^1^,^10^ Protein citrullination occurs in various physiological processes such as terminal differentiation of the epidermis, brain development, and regulation of gene expression via chromatin remodeling as well as in pathological conditions such as multiple sclerosis, psoriasis, Alzheimer disease, primary open-angle glaucoma, obstructive nephropathy and RA.^10^

Majority of RA cases are triggered by an autoimmune response to citrullinated proteins. These citrullinated proteins are believed to trigger an immune response by binding to HLA-DRB1 SE molecules on antigen presenting cells, leading to activation of pathogenic T and B cells, and ultimately promoting anti-CCP antibodies formation.^1^ The anti-CCP antibodies form immune complexes with these citrullinated proteins results in release of mediators of inflammation and joint destruction in RA.^10^,^11^

RA and chronic periodontitis share similar pathogenetic and immunologic mechanisms.^12^ Patients with Periodontal Disease (PD) were found to have an increased risk of developing RA and vice versa. PD were 2-fold more prevalent in patients with RA. Furthermore, the inflammatory cells and pro-inflammatory cytokines involve in chronic bone erosion in RA and chronic periodontal destruction in PD was found to be similar. The cytokines include interleukin-1 (IL-1), interleukin-6 (IL-6) and Tumour Necrosis alpha (TNF-α).^12^

For many years, Rheumatoid Factor (RF) was known to be the most important autoantibody and can be found in 60 to 80% of RA patients. However, RF is not specific for RA since it also can be present in chronic inflammatory conditions, viral infections as well as healthy individuals.^1^ RF displays a sensitivity of 70 to 80% and a specificity of 78% in established RA.^13^ On the other hand, anti-CCP shows a similar sensitivity (70 to 80%) but has a higher specificity (ranging from 88% to 98%) compared to RF, even in early stage of RA.^14^ Therefore, anti-CCP currently is considered to be a well-established diagnostic and prognostic marker for RA.^1^

Accumulating studies^11^,^15^,^16^ had shown that anti-CCP is found both in the periodontal tissues and the synovial tissues in RA patients, suggesting presence of association between anti-CCP, chronic periodontitis and RA. However, there is limited data regarding this association in the Malaysian population. Hence, this study was conducted to assess the presence of periodontal disease and whether the association with anti-CCP occurs among RA patients in local population.

## METHODS

This cross-sectional study was conducted at the Rheumatology Clinic and Dental Clinic of Hospital Universiti Sains Malaysia (USM), Kubang Kerian, Kelantan, Malaysia from July until October 2016. Forty-four RA patients aged 20 to 70 years old were able to be recruited and their periodontal status were examined. Patients with uncontrolled systemic diseases such as Systemic Lupus Erythematosus (SLE), diabetes mellitus, heart diseases, and blood disorders; inflammatory arthritis such a psoriatic arthritis, osteoarthritis, and gout; smokers, pregnant women and patients who were taking drugs influencing gum tissues were excluded. The diagnosis of RA was based on the American College of Rheumatology/European League against Rheumatism (ACR/EULAR).^17^

Informed written consents were obtained from all patients. Demographic data such as age, gender, ethnicity, duration of having diagnosed with RA were recorded. The study protocol was approved by Human Research and Ethics Committee, Universiti Sains Malaysia [(USM/JEPeM/16030121) Dated: 16^th^ June 2016] prior to data collection procedures.

### Oral Examination

A full-mouth periodontal examination was carried out on each subject to obtain the Plaque Score (PS), Gingivitis Score (GS), Periodontal Pocket Depth (PPD), and Clinical Attachment Loss (CAL). The examination was performed by using Michigan “O” periodontal probe with Williams marking at 1, 2, 3, 5, 7, 8, 9 and 10mm grading. The number of tooth loss was also recorded.

Disclosing agent was used to stain all tooth surfaces presence with plaque for assessment of PS. The periodontal probe is then used to examine the stained plaque on four surfaces of the tooth which are on mesial, distal, buccal/labial and lingual/palatal. The total number of tooth surfaces with stained plaque is counted; the sum is then divided by the number of all tooth surfaces available, and multiplied by 100 in order to establish the PS as a percentage.^18^ As for GS, periodontal probe and mouth mirror is used to detect Bleeding On Probing (BOP) on 4 surfaces of the tooth which are on mesial, distal, buccal/labial and lingual/palatal. The total number of scored tooth surfaces is counted; the sum is then divided by the number of all tooth surfaces in concern (including pontics and implants), and multiplied by 100 in order to establish the gingivitis score as a percentage. Meanwhile, PPD and CAL were measured on all six sites of the tooth namely mesiobuccal, midbuccal, distobuccal, mesiolingual, midlingual and distolingual. All measurements were recorded in the periodontal chart. The severity of periodontal status was defined as mild (1-2mm CAL) or moderate to severe (≥3 mm CAL).^19^

### Measurement of Anti-CCP Level

Five milliliters venous blood was collected from all participants for anti-CCP test. The measurement of anti-CCP level was performed according to the manufacturer’s protocol using the commercially available Enzyme Linked Immunosorbent Assay (ELISA) kit, AESKULISA (AESKU DIAGNOSTICS GmbH & Co. KG, Germany). The anti-CCP level of > 18U/ml was considered as positive.

### Statistical Analysis

Data obtained was analysed using statistical package for social sciences (SPSS) version 22.0 software. Demographic profile was expressed by descriptive analysis. Anti-CCP levels and periodontal parameters were described in mean and Standard Deviation (SD). Fisher’s Exact test was used for association between anti-CCP antibodies level and periodontal disease status based on clinical attachment level 19 as the expected count for category of periodontal status was less than 5. Significant level was set at *p*< 0.05 at 95% Confidence Interval (CI).

## RESULTS

Out of 44 patients, 84.1% were females with majority of them were Malays (84.1%). The mean age was 50.9 ± 13.8 (S, Dev) years old with the mean duration of RA was 8.8 ± 7.9 (S. Dev) years in which 47.7% of them had suffered from RA of more than five years as shown in Table-I. All patients were established diagnosed with RA and undergoing regular follow-up with Rheumatology Clinic, Hospital USM.

**Table-I:** Demographic characteristics of study subjects (n=44).

Characteristics	No, (%)	Mean ± S. Dev
Age (years)		50.9 ± 13.80
Gender		
Male	7 (15.9)	
Female	37 (84.1)	
Ethnicity		
Malay	37 (84.1)	
Chinese	7 (15.9)	
Duration of RA (years)		8.8 ± 7.9
≤ 5 years	23 (52.3)	
> 5 years	21 (47.7)	

RA: rheumatoid arthritis The anti-CCP antibody levels in RA patients are shown in Table-II. More than 50% of patients had positive anti-CCP with the mean level of anti-CCP was 180.0 (SD290.3).

The anti-CCP antibody levels in RA patients are shown in Table-II. More than 50% of patients had positive anti-CCP with the mean level of anti-CCP was 180.0 (SD290.3).

**Table-II:** Anti-CCP antibody levels of rheumatoid arthritis patients (n=44)

	No. (%)	Mean ± (S.Dev)
Anti-CCP level		180.0 ± 290.3
Negative (<18 Unit/mL)	21 (47.7)	
Positive (≥18 Unit/mL)	23 (52.3)	

Anti-CCP: Anti-cyclic citrullinated peptide antibodies.

Oral examination showed 27.3% had poor oral hygiene with plaque score more than 60%, 52.3% had generalized gingivitis (GS > 30%) and 20.5% had mean CAL of more than 3mm. However, none of the subjects presented with mean PPD of more than 3mm. It was also found that 36.4% of patients missing more than 10 teeth. The details of periodontal and dental status were shown in Table-III. Further analysis using Fisher’s Exact test showed that there was no significant association between the level of anti-CCP and periodontal status (p=0.20) (Table-IV).

**Table-III:** Periodontal status and anti-CCP levels of study subjects (n=44).

Variables	No. (%)	Mean ± S. Dev	Anti-CCP antibody levels (Unit/ml) Mean ± S. Dev
Plaque score (%)		48.5 ± 22.12	
Mild (≤25%)	8 (18.2)		70.36 ± 137.06
Moderate (>25-60%)	24 (54.5)		265.88 ± 348.92
Poor (>60%)	12 (27.3)		84.22 ± 167.51
Gingivitis score (%)		33.5 ± 23.18	
Localized gingivitis (≤30%)	21 (47.7)		219.91 ± 313.70
Generalized gingivitis (>30%)	23 (52.3)		145.07 ± 269.17
Periodontal pocket depth (mm)		1.9 ± 0.54	
≤3mm	42 (95.5)		188.08 ± 295.24
>3mm	2 (4.5)		27.50 ± 24.75
Clinical attachment loss (mm)		2.4 ± 1.01	
Mild (≤3mm)	35 (79.5)		212.67 ± 313.26
Moderate to severe (>3mm)	9 (20.5)		56.81 ± 119.03
Number of tooth loss		9.6 ± 7.53	
Loss more than 10 teeth	16 (36.4)		250 ± 365.13
Loss 10 teeth and less	28 (63.6)		141 ± 235.92

**Table-IV:** Association between the level of anti-CCP antibodies and the severity of periodontal disease based on CAL (n=44).

Severity of periodontal disease	n	Anti-CCP level		p-value^a^

		Negative (<18Unit/mL) No. (%)	Positive (≥18Unit/mL) No. (%)	
Mild (1-3mm CAL)	35	15 (42.9)	20 (57.1)	0.27
Moderate to severe (>3mm CAL)	9	6 (66.7)	3 (33.3)	

Anti-CCP: Anti-cyclic citrullinated peptide antibodies; CAL: clinical attachment loss, a Fisher’s Exact test.

## DISCUSSION

In this study, the mean age of RA patients was 50.9 years, with the youngest being 30 years old and oldest being 78 years old. This result is almost similar to studies done in Ipoh, Malaysia^20^ and UKM Medical Centre, Malaysia^5^ with the mean age of RA patients is 52.9 years and 51.0 years respectively. Majority of patients were females (84.1%) which indicates RA is mainly occur in middle age women with female preponderance as also demonstrated in several local studies in Malaysia.^5^,^21^,^22^ Besides, Malays were the most ethnic group had been recruited that reflects the demographic pattern of Malaysian population in which Malays is the major ethnic group followed by Chinese and other races.^5^

We found that 52.3% of subjects were positive for anti-CCP antibodies. Our finding is consistent with previous study by Snir and colleagues in 2010 which reported that anti-CCP antibodies were found in 55.0 to 69.0% of RA patients.^23^ However, previous study by Niewold *et al*. in 2007 discovered only 34.0 to 40.0% of the RA patients had positive anti-CCP results prior to the disease onset.^24^ Two previous local studies in Malaysia^21^,^22^ reported that the prevalence of Malaysian RA patients with positive anti-CCP antibodies were 80.4% and 68.0% respectively. Based on these findings, it is an established fact that most of RA patients have positive anti-CCP antibodies suggesting the possibility of breakdown of immune tolerance occur in RA patients leading to production of anti-CCP antibodies.

Plaque score is done to assess the oral hygiene status whereas gingivitis score reflects the inflammatory status of the gingiva. In the present study, majority of the patients (81.8%) were having fair to poor oral hygiene which is consistent to the presence of generalized gingivitis (52.3%).

Among them, 79.5% had mild form of periodontal disease with 1 to 3mm of CAL. This finding is almost similar to the survey done in Malaysia which demonstrated 96.7% of the adult population experienced periodontal disease.^25^

There were 36.4% of patients who were partially edentulous with missing more than 10 teeth. A study done among the United States population had stated that individuals diagnosed with RA are more susceptible to tooth loss.^15^ In addition, a systematic review by Kaur and co-workers^26^ also reported that increased tooth loss was more common in individuals with RA than in those without RA. However, such correlation was not determined in this present study thus, requires further investigation.

This study showed no significant association between anti-CCP level and the severity of periodontal disease based on CAL. This finding is consistent with a study in Korea in which they also found no significant association between levels of anti-CCP antibodies and severity of periodontitis.^27^ By contrast, in a study done in United States, RA patients who were positive for anti-CCP were more likely to have moderate to severe periodontitis (56.0%) than patients who were negative for anti-CCP (22.0%) (*p*=0.01).^28^ In addition, Harvey in 2012 reported positive RF or anti-CCP antibodies was associated with more severe periodontitis in RA patients compared to seronegative RA patients and control group.^29^ Nevertheless, this present study had involved only small number of sample size as its limitation. Hence, a further study with a larger sample size is recommended in order to achieve more conclusive findings.

## CONCLUSION

Most patients had positive anti-CCP with high mean level. Generalised gingivitis was prevalent in the selected RA patients. This finding reflects the important role of anti-CCP as a marker for disease activity in RA patients, however the association with periodontal status was not evident in this study. Nevertheless it is still beneficial to monitor patients’ oral hygiene to reduce gingivitis as well as to prevent unwanted disease progression and complications.
